# Increasing Contraceptive Use Through Free Family Planning Special Days in Poor Urban Areas in Francophone West Africa

**DOI:** 10.9745/GHSP-D-22-00227

**Published:** 2024-05-21

**Authors:** Mamadou Kandji, Hawa Talla, René Jean Firmin Nakoulma, Sujata Naik Bijou, Cheikh Ibrahima Diop, Josephat Avoce, Fatoumata Bamba, Fatimata Sow

**Affiliations:** a The Challenge Initiative, Francophone West Africa Hub, IntraHealth International, Dakar, Senegal.; b Clinton Health Access Initiative, Dakar, Senegal.; c IntraHealth International, Chapel Hill, NC, USA.; d Ministry of Health and Public Hygiene, Cote d'Ivoire.

## Abstract

**Introduction:**

In francophone West Africa (FWA), contraceptive uptake remains limited, often due to geographic, economic, and social barriers. With technical support from The Challenge Initiative (TCI), municipalities and health systems implemented Family Planning Special Days (FPSDs) to improve family planning (FP) uptake and reduce high unmet need. The FPSD intervention consisted of organizing free FP services on a monthly or quarterly basis over 2 to 5 consecutive days within health facilities or sites close to the population. These events helped to educate, inform, and mobilize the community around FP and improve geographic and financial access to FP services. We describe the process of implementing FPSDs in FWA countries and analyze the results.

**Methods:**

We used several techniques and data sources in our descriptive analysis, including document review of activity reports, analysis of health management information system data, and retrospective data collection on the profile of FPSD users and implementation costs.

**Results:**

Between July 2020 and June 2021, municipalities and health systems collaborated to hold 1,046 FPSDs in 452 health facilities in 10 FWA cities. This collaboration was made possible through the establishment of city-level management and coordination units composed of municipal, health system, and TCI focal points. In the 10 FWA cities, 181,792 people were made aware of the FPSDs and 71,669 contraceptive users were served. The overall cost of organizing the FPSDs was about 145382501 Central African CFA francs (US$252839), 35% of which came from the municipalities’ local financial contribution.

**Discussion:**

Results from our analysis showed that, with appropriate financial support from municipalities, the health system could offer high-quality free FP services. Nonetheless, there are still challenges to the sustainability of conducting FPSDs, including the availability of contraceptive products and continued financing of the strategy during system shocks such as the COVID-19 pandemic.

## Abstract (precis)

Through the support of local governments and The Challenge Initiative, Family Planning Special Days successfully facilitated the delivery of free family planning services to urban West African populations with high levels of poverty and unmet need.

## Teaser

**Key Findings:**

- Family Planning Special Days (FPSDs) reach new contraceptive users, particularly adolescents and youth, through a combination of free services, proximity, convenience, and awareness raising.
- During our study period, FPSDs in francophone West Africa provided free long-acting contraceptive methods to a majority of the women of reproductive age who chose a method.
- Municipalities paid for 35% of the total cost of FPSDs held during the study period.

**Key Implications:**

- Municipalities must allocate more financial resources to the health system to ensure the sustainability of conducting FPSDs.
- Other challenges to the sustainability of FPSDs include contraceptive stock-outs and continuity of family planning services during system shocks such as the COVID-19 pandemic.

## INTRODUCTION

Although health care access has concerned authorities in francophone West Africa (FWA) for many years, they have been focused primarily on the issue of geographical proximity. However, persistent low health care service utilization has highlighted the multiple issues limiting access, including financial barriers. This is particularly true in urban areas, where many people do not access care, particularly family planning (FP) services, despite proximity to health care facilities.^1^ FWA cities have a high prevalence of poverty, with widening gaps between classes, low contraceptive prevalence rates, and high unmet need (28%).^2^

Building on the demonstrated success of the Urban Reproductive Health Initiative, The Challenge Initiative (TCI) was established to accelerate contraceptive method use in poor urban areas. Since 2016, TCI FWA, led by IntraHealth International, has improved access to FP services for women of reproductive age (15–49 years) using high-impact interventions, including free Family Planning Special Days (FPSDs) adopted from the Urban Reproductive Health Initiative. Documentation of that project's interventions showed that for the free FPSDs, the targets were largely exceeded in terms of recruitment of new FP clients (104%) and couple-years protection (118%).^3^ The free FPSD approach was also tested in Mauritania as part of the U.S. Agency for International Development–funded Agir Pour La Planification Familiale project in 2017. Documentation of the approach there showed exceptional performance of the special days compared to routine days, with 5.5 more clients recruited and 7 more new clients recruited.^4^ FPSDs were also included on TCI University—an online platform housing the framework and tools associated with evidence-based FP and adolescent and youth sexual and reproductive health interventions.^5^

We describe how municipalities and the health system collaborated to implement FPSDs in FWA, the effectiveness in reaching women with FP services, associated costs, lessons learned, and recommendations to sustain and scale FPSDs.

## FPSD

FPSDs were an intervention organized by municipalities to provide free FP services on specific days in the community. Each FPSD event was held over 2 to 5 consecutive days in the health facility (fixed strategies) or in other sites close to people's homes (advanced or mobile strategies). This intervention was implemented in a regional context where the public health facilities, except for those in Burkina Faso, routinely charged fees for contraceptive products and services.

Through FPSDs, municipalities provided free FP services on specific days in the community at health facilities or in sites close to homes.

**Figure:**
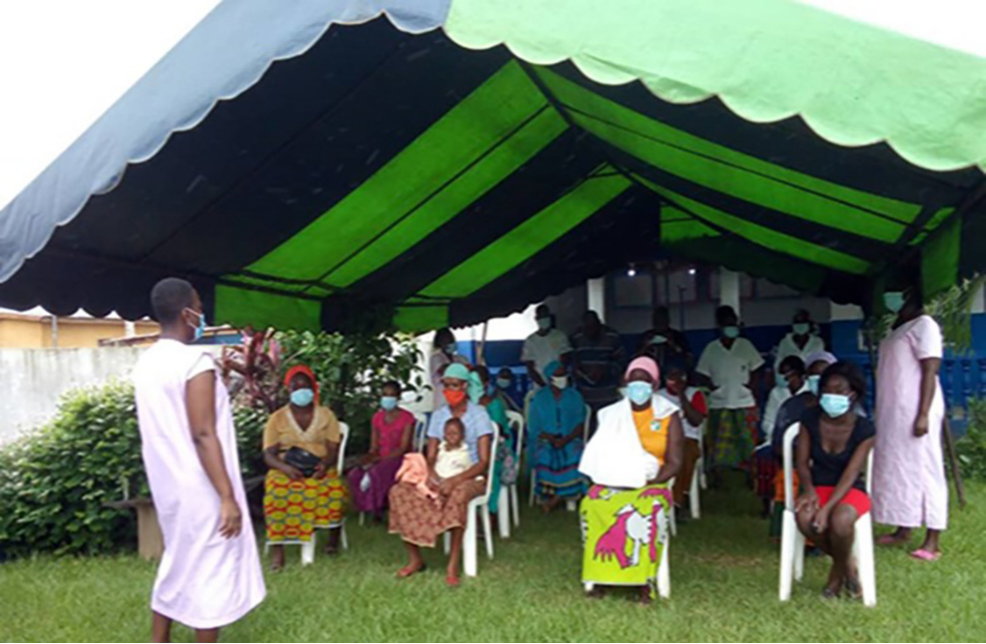
Community members attend a family planning sensitization session as part of a Family Planning Special Day intervention during the COVID-19 pandemic in Abobo, Abidjan, Côte d’Ivoire. © 2020 Kouakou Yao Degah Bienvenu Attoumbre/The Challenge Initiative Francophone West Africa–IntraHealth International

### Roles of Different Stakeholders

The cities supported by TCI FWA included FPSDs in their action plans to strengthen FP and adolescent and youth sexual and reproductive health. As part of the implementation of these plans, TCI, municipalities, and health systems created program management and coordination units (PMCUs) composed of government focal points from each municipality (including financial officers), health districts, and the regional health directorate. Each municipality signed bylaws establishing the PMCU and integrated it into the city's institutional set-up to ensure the PMCUs’ sustainability.

### Preparation and Resource Mobilization

The PMCU met monthly to plan activities and evaluate progress. Decisions were made on the following: locations; target population (typically poor, disadvantaged populations); number of FPSDs; required personnel, logistics, and contraceptive products and consumables; and budget. The terms of reference for the FPSDs and the budget request were submitted to the municipality for fund disbursement. The TCI focal point worked with the PMCU to ensure that the city action plan, including FPSDs, was included in health district annual work plans.

Preparatory meetings were organized between the health system, the municipality, and the community (represented by a community relay or the neighborhood delegate) to estimate the need for contraceptive products and consumables, prepare management tools (e.g., forms, registers, cards, etc.), mobilize logistics (e.g., vehicles, tarpaulins, chairs, and sound system), and identify qualified health system personnel (doctors, midwives, and nurses) and support staff (e.g., community health agents, drivers, maintenance agents, etc.). Finally, an advance site visit was conducted to verify that the premises (health huts, town halls, and sociocultural centers) were adequate.

### Marketing and Communication

A communication plan for FPSDs was developed and implemented for 4 to 5 days (2 to 3 days before the activity and the first 2 days of the activity). Messages were disseminated through mass media (e.g., posters, banners, radio, television, social networks, and newspapers) and town criers. Community volunteers held informational meetings with religious, traditional, and community leaders and conducted home visits, during which they gave referral cards with information about FPSDs.

#### Conduct of the FPSDs

FPSDs were an outreach service conducted in health facilities (fixed strategies) or in other sites close to where people live (advanced or mobile strategies). During FPSDs, after health providers provided educational talks, women were directed to consultation rooms where they were counseled, offered their FP method choice, and given follow-up visit advice. For follow-up and management of complications, clients had to return to the health facility. If the site staff could not manage the complication, the client was referred to the district hospital using a standard referral form. The FPSDs were conducted under the supervision of the health system district management team to ensure that the services offered were of high quality and the activity ran smoothly. In 2021, FPSDs were implemented in 10 cities across 5 FWA countries (Table 1).

**TABLE 1.** TCI-Supported Cities Implementing Family Planning Special Days in Francophone West Africa, July 2020–June 2021

**Country**	**City**	**Population, No.**	**Women of Reproductive Age, No.**	**Participating Health Facilities, No.**	**Contraceptive Prevalence Rates per Country per Region, %** ^6^ ^–^ ^10^	**Unmet Need Rate per Country per Region, %** ^6^ ^–^ ^10^
Benin	Abomey Calavi	1,480,982	355,436	63	9.9	37.5
	UCOZ	1,228,667	294,880	116	13.9	31.2
	Cotonou	1,108,320	265,997	73	19.8	35
Burkina Faso	Koudougou	183,332	408,207	67	31.8	16.3
	Ouagadougou	2,415,266	876,104	153	38	16.9
Côte d'Ivoire	Abidjan	5,616,633	2,684,751	155	21.7	21.9
	Bouaké	931,851	145,953	32	21.9	18.6
Niger	Niamey	1,565,056	375,613	62	26.2	17.7
Senegal	Nioro	365,534	87,728	51	23.6	22.6
	Ziguinchor	319,425	79,856	109	22.6	22.7
Total		15,215,066	5,574,525	881		

Abbreviations: TCI, The Challenge Initiative; UCOZ, Union of Zou Municipalities.

During FPSDs, health providers provide educational talks after which women are directed to consultation rooms where they are counseled, offered their FP method choice, and given follow-up visit advice.

**Figure:**
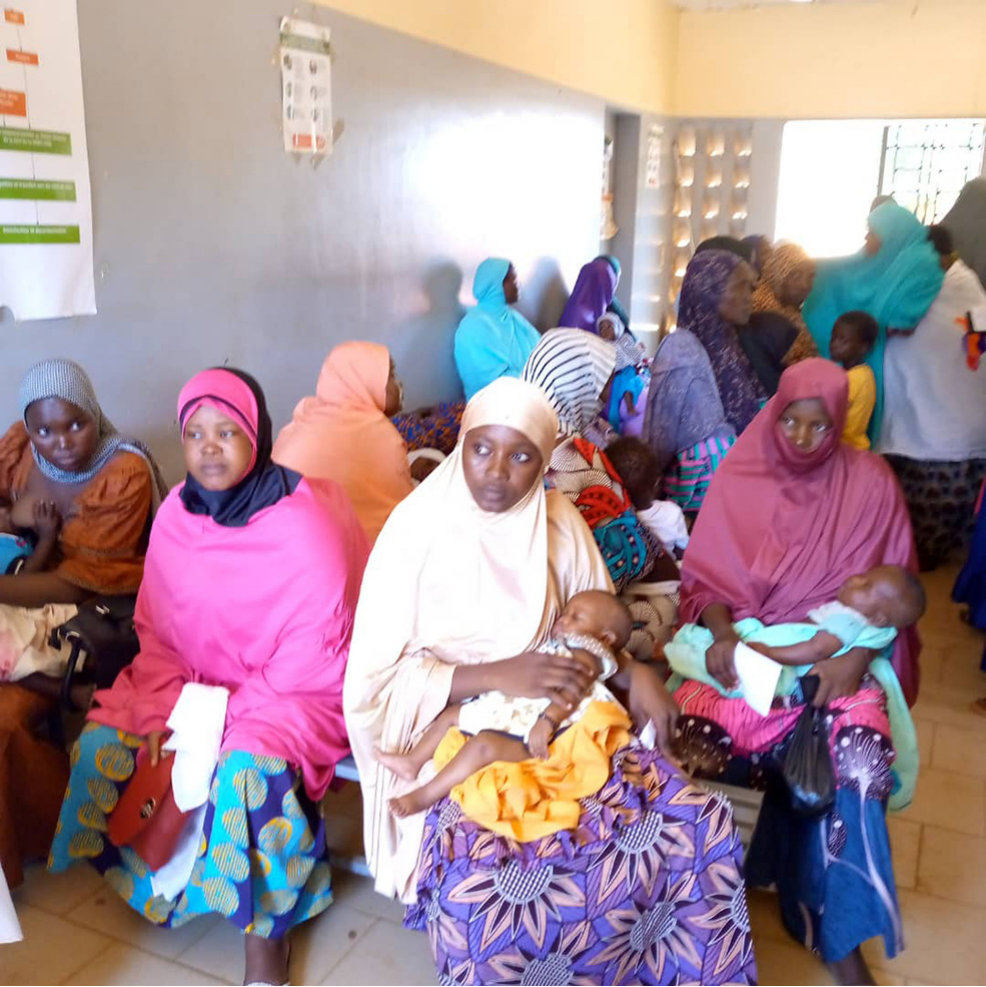
Women attend a Family Planning Special Day in Niamey, Niger. © 2021 Fatimata Sow/The Challenge Initiative Francophone West Africa–IntraHealth International

### Data Management

FPSD management tools were the same as those used in the health system, including appointment cards, FP cards, FP registers, referral and counter-referral forms, and an FPSD data summary form. Daily reports and a summary report were prepared and sent to the district, which were then sent to TCI. The summary report included the location, populations sensitized to the FPSDs, health personnel mobilized, FPSD service users, and quantities of products distributed. Each month, health facilities integrated these data into their own monthly report. The district data manager entered these data into TCI FWA’s database and archives the collection sheets and reports.

## METHODS

This descriptive analysis, which covers the period from July 2020 to June 2021, was based on document review of activity reports, analysis of health management information system data, and retrospective data collection of implementation costs. All health facilities that organized FPSDs with data entered in the project database from July 2020 to June 2021 were selected in the 36 districts supported by TCI. We developed a data collection form in Epi Info 7 to enter and analyze data on finances from all FPSD expenditure reports of all cities during the study period. Table 2 includes the indicators used for this analysis.

**TABLE 2.** Indicators Used in Analysis of Family Planning Special Days in Francophone West Africa

**Indicator**	**Definition**	**Data Source**
Average number of days of FPSDs held per facility	The total number of days of FPSDs divided by the total number of facilities that held FPSDs	Project data
Number of people sensitized on FPSDs	The number of people (men and women) who were informed about the FPSDs	HMIS data
Total number of users recruited during the FPSDs	The total number of new and existing FP users who were served during the FPSDs	HMIS data
Annual client volume for FPSDs	The total number of users (new and existing) adjusted for: Seasonality (rolling sums) with 12-month rolling sum (July 2020–June 2021) for each contraceptive method Revisits (for short-acting methods) using inverse CYP coefficients	HMIS data
Daily FPSDs yield or average number of users recruited per day	The total number of users recruited at FPSDs out of the total number of days for FPSDs	HMIS data
Total number of adolescents and youth recruited at FPSDs	The total number of new and existing FP users, aged 15–24 years, who were served at FPSDs	HMIS data
Average cost of FPSDs	The overall amount spent on FPSDs divided by the total number days of FPSDs conducted	Project data
Average operational cost of recruiting a female FP user through FPSDs	The total amount spent on FPSDs divided by the total number of FP users recruited through FPSDs	Project data

Abbreviations: CYP, couple-years protection; FP, family planning; FPSD, Family Planning Special Day; HMIS, health management information system.

### Ethical Approval

This analysis is consistent with international standards for the ethical conduct of research. No personal information was collected; key informant questions related only to the normal course of the informants’ work.

## RESULTS

### Characteristics of Health Facilities Implementing FPSDs

**FIGURE 1:**
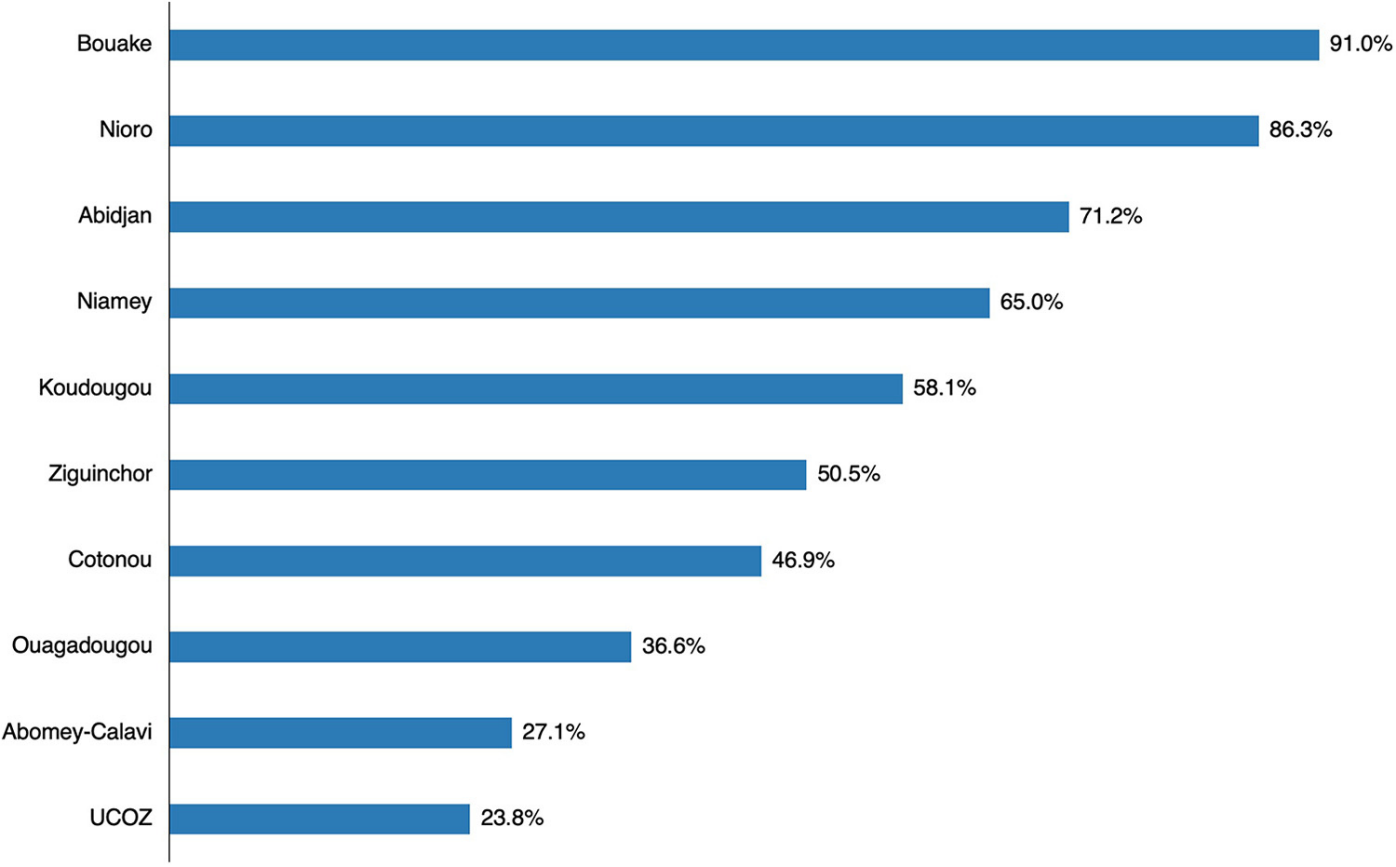
Percentage of The Challenge Initiative–Supported Health Facilities That Organized Family Planning Special Days in Francophone West Africa by City, July 2020–June 2021 Abbreviation: UCOZ, Union of Zou Municipalities.

During the period analyzed, 1,046 FPSDs were conducted in 452 health facilities. More than half (51.2%) of TCI partner health facilities organized FPSDs, with the percentage varying greatly by city, from 23.8% in the Union of Zou Municipalities (UCOZ), Benin, to 91% in Bouake, Côte d'Ivoire (Figure 1).

Of the health facilities that organized FPSDs, 94% were public and 6% were private. The average number of days of FPSDs organized per health facility across all cities was 2.3, with Bouake holding the highest number of days (5.1) (Table 3).

**TABLE 3:** Average Number of Family Planning Special Days in Francophone West Africa per Health Facility in Cities, July 2020–June 2021

**Country**	**City**	**Health Facilities That Organized FPSDs, No.**	**Total FPSDs Organized, No.**	**Average FPSDs Organized per Health Facility, No.**
Côte d'Ivoire	Abidjan	42	56	1.3
	Bouake	15	77	5.1
	Subtotal	57	133	2.3
Benin	UCOZ	76	166	2.2
	Abomey-Calavi	15	46	3.1
	Cotonou	52	173	3.3
	Subtotal	143	385	2.7
Niger	Niamey	36	82	2.3
Burkina Faso	Ouagadougou	56	95	1.7
	Koudougou	61	97	1.6
	Subtotal	117	192	1.6
Senegal	Ziguinchor	55	108	2.0
	Nioro	44	146	3.3
	Subtotal	99	254	2.6
Total		452	1,046	2.3

Abbreviations: FPSD, Family Planning Special Day; UCOZ, Union of Zou Municipalities.

### Characteristics of Health Personnel Mobilized for FPSDs

**FIGURE 2:**
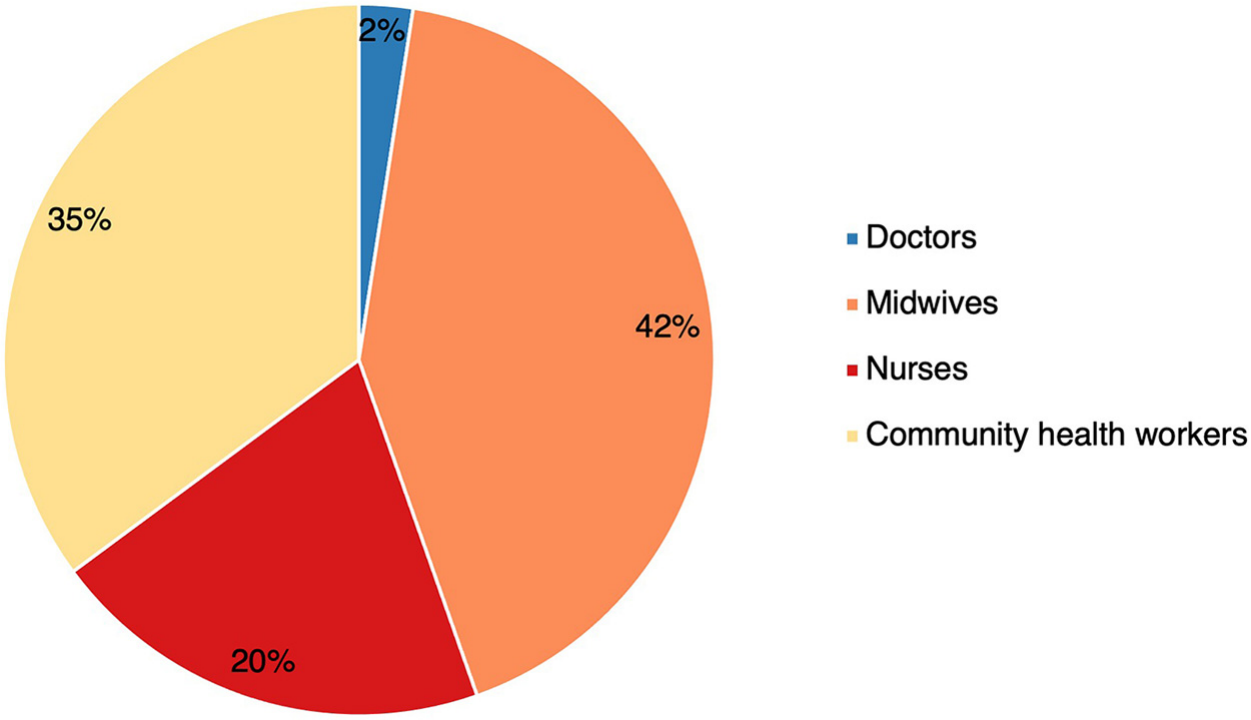
Percentage of Health Personnel Mobilized for Family Planning Special Days in Francophone West Africa by Category, July 2020–June 2021 (N=525)

**FIGURE 3:**
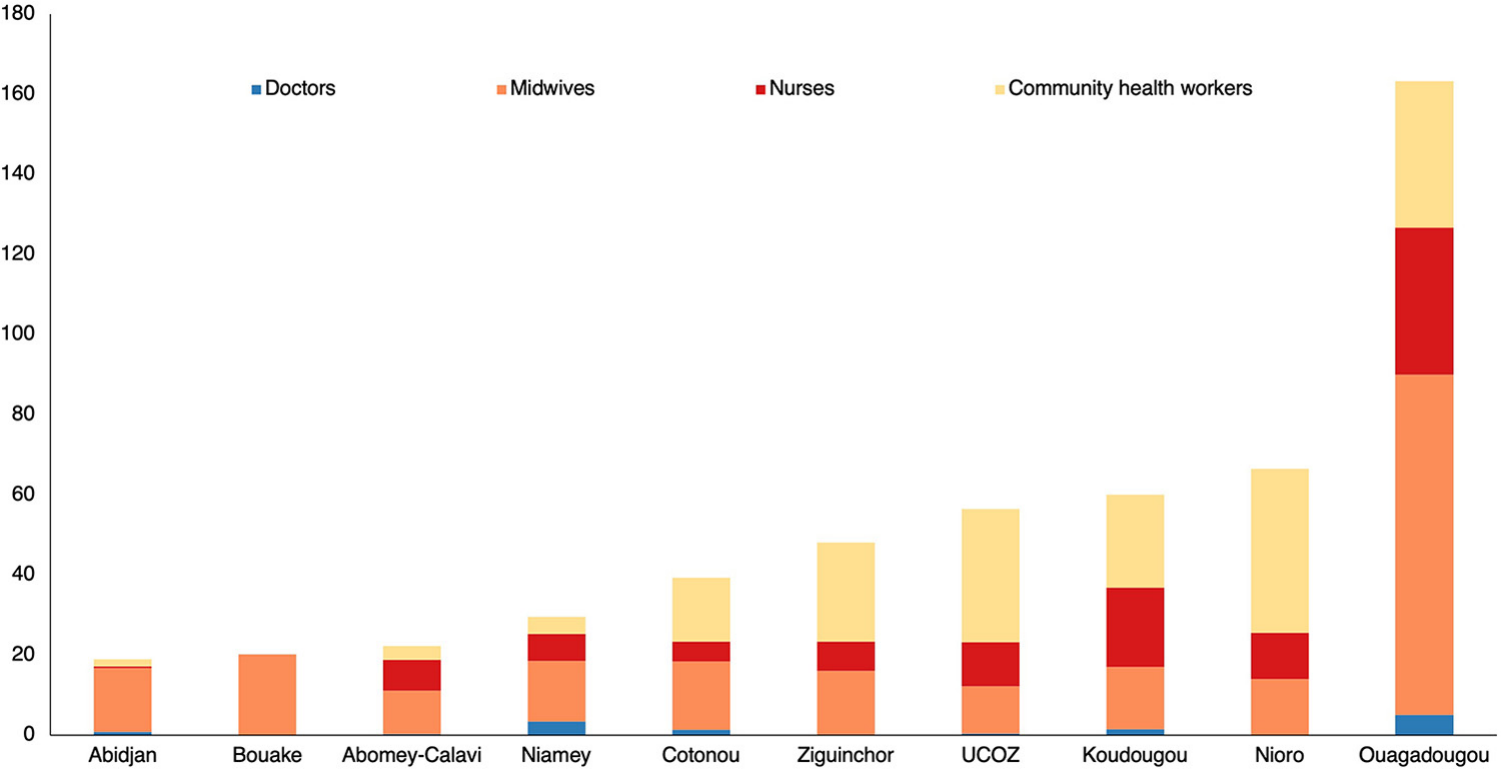
Number of Health Personnel Mobilized for Family Planning Special Days in Francophone West Africa by Category and City, July 2020–June 2021 Abbreviation: UCOZ, Union of Zou Municipalities.

FPSDs were primarily conducted by midwives, with an average of 221 (42%) midwives per month, followed by community health workers, nurses, and doctors (Figure 2). This was expected because midwives generally offered FP services with support from community health workers and nurses. Physicians generally supervised the conduct of the FPSDs. Ouagadougou mobilized the largest number of staff, while Bouake mobilized only midwives (Figure 3).

### Sensitization of Populations About the FPSDs

During the FPSDs, 583 community volunteers (or health workers) conducted 22,960 awareness activities. A total of 1,638 media broadcasts reached 181,792 people, 17% of whom were men. Among people made aware of the FPSDs, the majority were aged 20–24 years (53%) compared to those aged 25–49 years (44%). Niamey, UCOZ, and Ouagadougou reached the largest number of people during the FPSDs. Of those reached, 54,619 (30%) were referred to the FPSDs (data not shown).

### Service Delivery Results

From July 2020 to June 2021, FPSDs enabled the provision of services to 71,669 users of modern contraceptive methods. Almost half (46%) of FPSD service users were aged 15–24 years. The most used contraceptive methods were implants, followed by injectables and pills, with 34.3%, 33.9%, and 15.2%, respectively (Table 4). A similar percentage of long-acting methods was found with the implementation of the FPSDs in Mauritania.^4^ This distribution may be explained in part by the lack of fees during FPSD days compared with the high price of these methods on non-FPSD days. Long-acting methods are generally more expensive than other methods on non-FPSD days in health facilities. For example, in Côte d'Ivoire, the cost of routine service delivery for implant insertion, including consumables, is 3000 Central African CFA francs (FCFA) (US$5), FCFA1000 (US$1.70) for injectables, and FCFA200 ($0.34) for pills, while in Senegal the costs for the same services are lower, at FCFA1500 (US$2.60) for implants, FCFA200 (US$0.34) for injectables, and FCFA100 (US$0.17) for pills.

**TABLE 4.** Beneficiaries of Family Planning Special Days in Francophone West Africa by Age Group and Method Type, July 2020–June 2021

Type of ContraceptiveMethod	Users Aged 15–19 Years, No.		Users Aged 20–24 Years, No.		Users Aged 25–49 Years, No.		Total Users, No. (%)
	New	Existing	New	Existing	New	Existing	
Implants	2,926	712	5,243	2,135	8,708	4,833	24,557 (34.3)
Injectable	2,035	1,032	4,000	3,532	6,285	7,414	24,298 (33.9)
Pills	1,058	817	1,588	1,954	2,015	3,433	10,865 (15.2)
Condoms	1,817	656	1,603	897	1,487	1,172	7,632 (10.6)
IUD	188	74	567	241	1,885	1,195	4,150 (5.8)
Emergency contraceptive	20	14	42	25	34	32	167 (0.2)
Total	8,044	3,305	13,043	8,784	20,414	18,079	71,669 (100)

Abbreviation: IUD, intrauterine device.

Across all age groups, 58% of beneficiaries were new contraceptive users. This percentage was much higher among adolescents and youth, who represented 51% of new contraceptive users. This finding suggests that the FPSD intervention mobilized new users through a combination of free services, proximity, convenience, and awareness raising.

Our findings suggest that the FPSD intervention mobilized new users through a combination of free services, proximity, convenience, and awareness raising.

These results were adjusted for seasonality and the couple-years protection for short-acting method users to calculate the annual client volume of 34,061 users (Table 5). Method mix analysis showed that 78% of these users adopted long-acting methods and 22% chose short-acting methods from July 2020 to June 2021. Among users choosing long-acting methods, 43% were adolescents and youth.

**TABLE 5.** Annual Family Planning Client Volume During Family Planning Special Days in Francophone West Africa by Age Group and Method Category, July 2020–June 2021

**Contraceptive Method**	**Users Aged 15–19 Years, No.**	**Users Aged 20–24 Years, No.**	**Users Aged 25–49 Years, No.**	**Total Users, No. (%)**
Long-acting	3,587	7,606	15,227	26,420 (78)
Short-acting	1,188	2,404	4,049	7,641 (22)
Total	4,775	10,010	19,276	34,061 (100)

The average number of total users recruited per day during FPSDs (daily output) was 68. This is much higher than the daily output found in Mauritania, which was only 28 users recruited on average per day.^4^ Abidjan, with an average of 222 total users recruited per day, had the highest daily output, followed by UCOZ (99 users) and Niamey (97 users) (Table 6). These differences can be explained by several factors: (1) the size of the population (the city of Abidjan has the largest population of any city, followed by Ouagadougou, Niamey, Abomey Calavi, and UCOZ); (2) the cost of routine FP services, as mentioned above; and (3) the level of unmet need, which is highest in Benin (32%), Côte d’Ivoire (22%), and Senegal (22%).^6^^–^^10^

**TABLE 6.** Daily Performance of Family Planning Special Days by City in Francophone West Africa, July 2020–June 2021

**Country**	**City**	**Total Users Recruited at FPSDs, No.**	**Total FPSDs, No.**	**Average Users Recruited per FPSD, No.**	**Total Providers, No.**	**Average Users Recruited per FPSD per Provider, No.**
Côte d'Ivoire	Abidjan	12,450	56	222	17	13.1
	Bouake	5,850	77	76	20	3.8
	Subtotal	18,300	133	138	37	3.7
Benin	UCOZ	16,454	166	99	23	4.3
	Abomey-Calavi	3,784	46	82	19	4.3
	Cotonou	4,131	173	24	23	1.0
	Subtotal	24,369	385	63	65	1.0
Niger	Niamey	7,947	82	97	25	3.9
Burkina Faso	Ouagadougou	8,290	95	87	127	0.7
	Koudougou	2,065	97	21	37	0.6
	Subtotal	10,355	192	54	164	0.3
Senegal	Ziguinchor	5,338	108	49	23	2.1
	Nioro	5,360	146	37	26	1.4
	Subtotal	10,698	254	42	49	0.9
Total		71,669	1,046	68	340	0.2

Abbreviations: FPSD, Family Planning Special Day; UCOZ, Union of Zou Municipalities.

The number of health facilities that organized FPSDs and the number of days of FPSDs organized did not have a substantial influence on the performance of FPSDs. Abidjan, which organized 56 days of FPSDs in 42 health facilities, had a higher yield than Koudougou, Cotonou, Nioro, or Ziguinchor, which organized more days of FPSDs with slightly more health facilities.

In addition, the overall average number of clients served per day per provider (doctors, midwives, and nurses) was 0.2, with significant variations by city. The city of Abidjan had the highest number of clients served per day per provider (13.1), and the cities of Koudougou and Ouagadougou had the lowest number of clients served per provider (0.7) (Table 6). The low number of users served by providers in certain cities (Cotonou, Nioro, or Ziguionchor) may reflect low attendance on special days, often due to a lack of preparation (e.g., low communication). For the cities of Burkina Faso, it could be linked to the country's context, which included free family planning services.

### Implementation Costs

Overall, FPSDs in FWA cost FCFA145382501 (US$252839). Contributions came mainly from municipalities (FCFA51223176 [34.9%]) and TCI FWA (FCFA94159325 [64.8%]), with the remaining balance from health facility management committees made up of community members. Niamey, which bore 53% of the expenses (FCFA11735400), was the only city that spent more than TCI. Apart from Niamey, Cotonou, Abomey Calavi, Abidjan, and Bouaké had contributions that exceeded the average, with FCFA5046800 (44%), FCFA2279720 (41%), FCFA9671200 (39%), and FCFA6090000 (39%), respectively (Table 7). Ouagadougou and Koudougou had the lowest contributions because contraceptive products were free in Burkina Faso, so expenditures were only made in cases of shortages of contraceptive products or consumables.

**TABLE 7.** Distribution of Financial Contribution^a^ to Family Planning Special Days in Francophone West Africa by City and Source, July 2020–June 2021

**Country**	**City**	**City Contribution, FCFA (%)**	**TCI Contribution, FCFA (%)**	**Total Contribution, FCFA**
Côte d'Ivoire	Abidjan	9671200 (39)	15274000 (61)	24945200
	Bouake	6090000 (39)	9450000 (61)	15540000
	Subtotal	15761200 (39)	24724000 (61)	40485200
Benin	UCOZ	4500000 (32)	9652350 (68)	14152350
	Abomey-Calavi	2279720 (41)	3343200 (59)	5622920
	Cotonou	5046800 (44)	6474400 (56)	11521200
	Subtotal	11826520 (38)	19469950 (62)	31296470
Niger	Niamey	11735400 (53)	10350000 (47)	22085400
Burkina Faso	Ouagadougou	850000 (5)	15550000 (95)	16400000
	Koudougou	1539991 (23)	5050000 (77)	6589991
	Subtotal	2389991 (10)	20600000 (90)	22989991
Senegal	Ziguinchor	6663265 (37)	11585375 (63)	18248640
	Nioro	2846800 (28)	7430000 (72)	10276800
	Subtotal	9510065 (33)	19015375 (67)	28525440
Total		51223176 (35)	94159325 (65)	145382501

Abbreviations: FCFA, West African CFA franc; TCI, The Challenge Initiative; UCOZ, Union of Zou Municipalities.

a 611 West African CFA francs=US$1.

**FIGURE 4:**
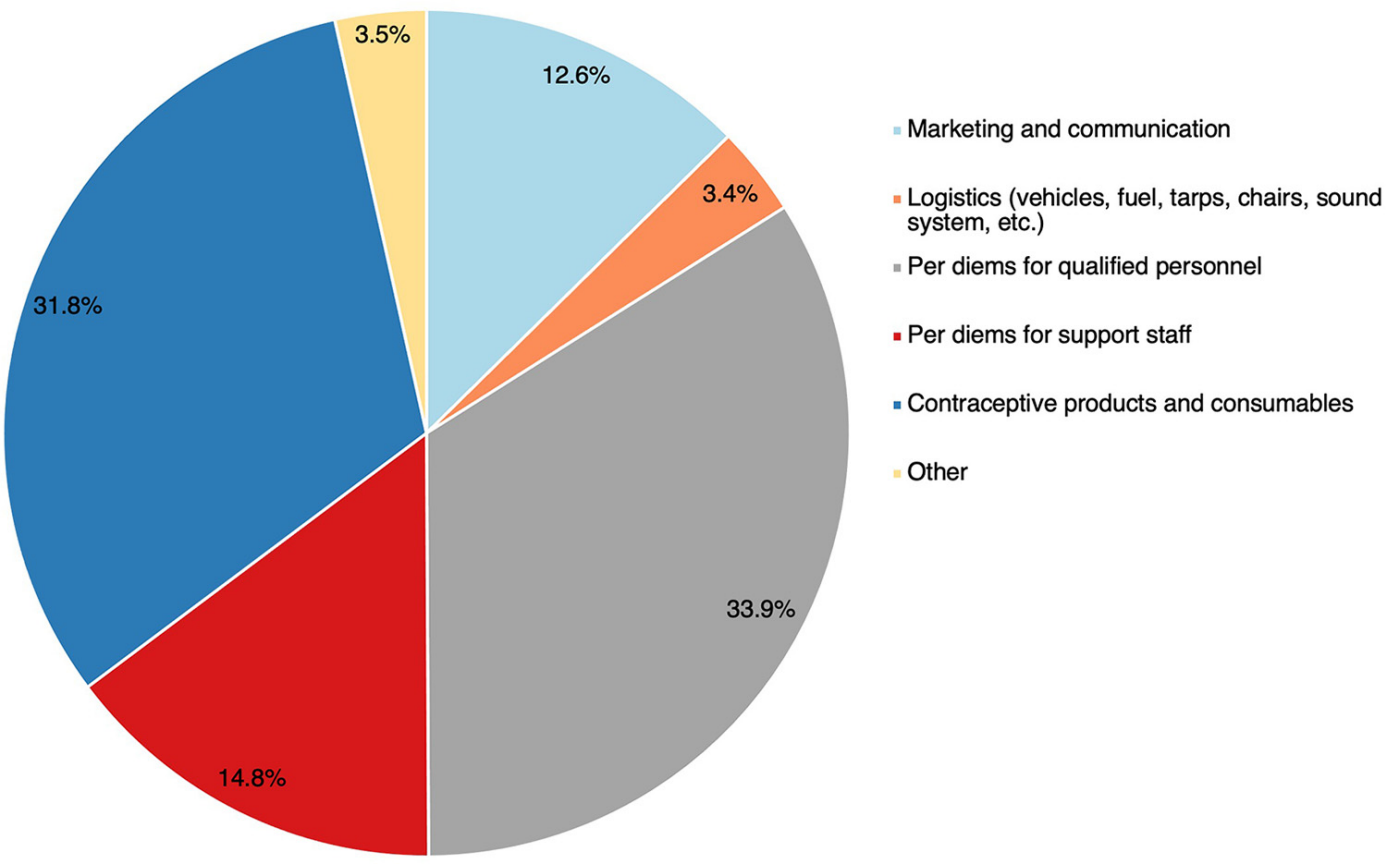
Expense Categories for Family Planning Special Days in Francophone West Africa, July 2020–June 2021

TCI’s expenses supported marketing and communication, logistics, and staff per diems. The bulk of the municipalities' contribution was for the purchase of contraceptive products (90.8%). Per diems for personnel represented 33.8% of expenses, followed by contraceptive products (31.7%) (Figure 4).

The average cost per day of organizing an FPSD event in FWA was FCFA138989 (US$242). The highest average costs for 1 FPSD day were in Abidjan, Bouake, and Niamey. Cotonou and Koudougou had the lowest average cost per day of FPSDs (Table 8). Differences in per diem rates and the categories of personnel mobilized for FPSDs, especially in Abidjan and Bouake, where nearly 85% of the personnel were midwives, could explain this variation.

**TABLE 8.** Average Cost per Day of Family Planning Special Days by City, July 2020–June 2021

**City**	**Total Cost of FPSDs, FCFA** ^a^	**Total FPSDs, No.**	**Users Recruited Through FPSDs, No.**	**Average Cost of FPSDs, FCFA (US$)**	**Average Cost per User, FCFA (US$)**
Abidjan	24945200	56	12,450	445450 (775)	2004 (3)
Bouake	15540000	77	5,850	201818 (351)	2656 (5)
Abomey-Calavi	5622920	46	3,784	122237 (213)	1486 (3)
UCOZ	14152350	166	16,454	85255 (148)	860 (1)
Cotonou	11521200	173	4,131	66596 (116)	2789 (5)
Niamey	22085400	82	7,947	269334 (468)	2779 (5)
Ouagadougou	16400000	95	8,290	172631 (300)	1978 (3)
Koudougou	6589991	97	2,065	67938 (118)	3191 (6)
Nioro	10276800	146	5,360	70389 (122)	1917 (3)
Ziguinchor	18248640	108	5,338	168968 (295)	3419 (6)
Total	145382501	1046	71,669	138989 (242)	2029 (4)

Abbreviations: FCFA, West African CFA franc; FP, family planning; FPSD, Family Planning Special Day; UCOZ, Union of Zou Municipalities.

a 611 West African CFA francs=US$1.

The average cost of recruiting 1 FP user was FCFA2029 (US$4) from July 2020 to June 2021. This average cost varied from FCFA860 (US$1) for UCOZ to FCFA3419 (US$6) for Ziguinchor (Table 8). Differences in the performance of cities in recruiting FP users at the FPSDs were not related to the amount of money spent. Indeed, Koudougou and Ziguinchor cities had the highest average cost of recruiting an FP user compared to Abidjan and UCOZ, which were more successful in terms of users recruited and had lower average costs per FP user. In terms of couple-years protection, the average cost of a user for FPSDs was FCFA4268 (US$7).

## LESSONS LEARNED

- **FPSDs improved geographical and financial access to contraception for women of reproductive age.** With the provision of free FP services, FPSDs enabled 71,669 women of reproductive age to access modern contraceptive methods in urban areas of FWA, with an annual client volume of 34,061. In Mauritania, the number of clients served per day during FPSDs was 5.5 higher than on the routine fee-for-service basis, with 6 new clients and 21 existing clients.^4^ FPSDs helped remove economic barriers that prevent some women from accessing contraceptive methods, particularly in poor urban areas.
- **FPSDs provided an opportunity for clients to benefit from free long-acting methods**. Of the total client volume during the FPSDs, a majority (78%) of users adopted long-term methods and only 22% adopted short-acting methods. A similar trend exists almost everywhere in sub-Saharan Africa, where implant use has increased substantially and evenly across almost all sociodemographic categories.^11^
- **FPSDs strengthened collaboration between stakeholders.** We noted substantial ownership by the municipalities and the health system, which set up the PMCUs. This body strengthened collaborations, bringing municipalities closer to the health system. This collaboration involved several actors: doctors (2%), midwives (42%), and nurses (20%); community health workers (35%); municipal representatives; and the general population.
- **FPSDs were a highly cost-effective intervention.** Providing comprehensive, high-quality contraceptive services to the growing number of women who need modern had important cost implications.^12^ The average cost of organizing 1 FPSD day was FCFA138989 (US$242), and the average cost of recruiting an FP user was FCFA2029 (US$4). In 2012, the average annual cost per user for all modern methods in the developing world was estimated at US$6.15 and for the Africa region at US$11.26.^12^

FPSDs help remove economic barriers that prevent some women from accessing contraceptive methods, particularly in poor urban areas.

## RECOMMENDATIONS

The promising results of FPSDs in FWA support the following recommendations.

- Ministries of health must make contraceptive supplies and consumables available to significantly reduce the costs associated with implementing this intervention and allow municipalities to cover the remaining costs, such as per diems and logistics.
- Ministries of health should include FPSDs in their FP strategic plans to enable health districts to include them in their annual work plans.
- Members of the coordination and management units, along with advocacy groups, must advocate with municipalities to include a substantial budget line for FP in their annual budgets to ensure the sustainability of this intervention.
- The Ministry of Health should build the FP communication capacity of community relays and providers to strengthen activities to help remove barriers to FP, create or increase demand for FP, and reinforce positive values and norms.^13^

## CONCLUSION

FPSDs could improve geographic and financial access to FP services. The results of the FPSDs in francophone West Africa were encouraging, despite the COVID-19 pandemic, because of the financial support of the participating municipalities and TCI. Successful implementation requires coordination and engagement of all stakeholders. These results should spur municipalities and health systems to institutionalize the FPSD intervention by integrating it into country policies and norms, increasing availability of contraceptive products and consumables, and continuing to provide financing.

## Supplementary Material

FR_GHSP-D-22-00227.pdf

## Notes

**Peer Reviewed**

**First published online:** February 13, 2023.
