# Supplementary material for: Increasing Contraceptive Use Through Free Family Planning Special Days in Poor Urban Areas in Francophone West Africa
Source: Glob Health Sci Pract. 2024 May 21;12(Suppl 2):e2200227. doi: 10.9745/GHSP-D-22-00227 (PMC11111110; doi:10.9745/GHSP-D-22-00227)
Supplement: FR_GHSP-D-22-00227.pdf [file FR_GHSP-D-22-00227.pdf]

RAPPORT D'ACTIVITÉS DE TERRAIN

Accroître l'utilisation des contraceptifs dans le cadre des Journées spéciales de planification familiale dans les zones urbaines en Afrique de l'Ouest francophone

Mamadou Kandji,[a] Hawa Talla,[b] René Jean Firmin Nakoulma,[a] Sujata Naik Bijou,[c] Cheikh Ibrahima Diop,[a] Josephat Avoce,[a] Fatoumata Bamba,[d] Fatimata Sow[a]

Principales conclusions

- Les Journées spéciales de planification familiale (JSPF) permettent de toucher les nouveaux utilisateurs de contraceptifs, en particulier les adolescents et les jeunes, grâce à une combinaison de services gratuits, de proximité, de commodité et de sensibilisation.
- Tout au long de la période d'étude, les JSPF en Afrique de l'Ouest francophone ont permis de fournir gratuitement des méthodes contraceptives à longue durée d'action à une majorité de femmes en âge de procréer ayant choisi une des méthodes de contraception.
- Les municipalités ont pris en charge 35 % du coût total des JSPF organisées pendant la période d'étude

Implications clés

- Les municipalités doivent allouer davantage de ressources financières au système de santé pour garantir la durabilité des JSPF.
- Parmi les autres défis à relever pour assurer la durabilité des JSPF figurent les ruptures de stock de contraceptifs et la continuité des services de planification familiale en situation de crise sanitaire affectant le système de santé, telle que lors de la pandémie de la COVID.

RÉSUMÉ

Introduction : En Afrique de l'Ouest francophone (AOF), l'adoption des contraceptifs reste limitée, et ceci très souvent en raison de barrières géographiques, économiques et sociales. Avec l'appui technique de The Challenge Initiative (TCI), les municipalités et le système de santé ont mis sur pied les Journées spéciales de planification familiale (JSPF) afin d'améliorer l'adoption de la planification familiale (PF) et de réduire le niveau élevé des besoins non satisfaits. L'intervention menée dans le cadre des JSPF consistait à offrir des services gratuits de PF tous les mois ou tous les trimestres sur une durée de 2 à 5 jours consécutifs dans les établissements de santé ou dans des sites proches des populations. Les différentes activités ainsi menées ont permis d'éduquer, d'informer et de mobiliser la communauté autour de la PF et d'améliorer l'accès aux services de PF, tant sur le plan géographique que sur le plan financier. Nous décrivons le processus de mise en œuvre des JSPF dans les pays de l'AOF, puis nous en analysons les résultats.

Méthodes : Nous avons utilisé plusieurs techniques et sources de données dans notre analyse descriptive, notamment l'examen des rapports d'activité, l'analyse des données du système d'informations sanitaires et la collecte de données rétrospectives sur le profil des utilisateurs des JSPF et les coûts d'exécution.

Résultats : Entre juillet 2020 et juin 2021, les municipalités et le système de santé ont, dans un esprit de collaboration, organisé 1 046 JSPF dans 452 établissements de santé de 10 villes en Afrique de l'Ouest francophone. Cette collaboration a pu être effective grâce à la mise en place d'unités de gestion et de coordination au niveau des villes, composées de points focaux issus des municipalités, du système de santé et de TCI. Dans ces 10 villes, 181 792 personnes ont été sensibilisées sur les JSPF et 71 669 utilisatrices de contraceptifs ont été servies. Le coût global de l'organisation des JSPF était d'environ 145 382 501 FCFA (252 839 USD), dont 35% provenaient de la contribution financière locale des municipalités.

Discussion : Les résultats de notre analyse ont montré qu'avec un soutien financier approprié de la part des municipalités, le système de santé pouvait offrir des services de PF gratuits de haute qualité. Néanmoins, il reste des défis à relever pour assurer la durabilité des JSPF, notamment en ce qui concerne la disponibilité des produits contraceptifs et la poursuite du financement de la stratégie en situation de crise sanitaire affectant le système de santé, telle que lors de la pandémie de la COVID.

INTRODUCTION

[a] The Challenge Initiative, Hub Afrique de l'Ouest francophone, IntraHealth International, Dakar, Senegal.
[b] Clinton Health Access Initiative, Dakar, Senegal.
[c] IntraHealth International, Chapel Hill, NC, USA.
[d] Ministère de la santé et de l'hygiène publique, Côte d'Ivoire.
Correspondence à Mamadou Kandji (mkandji@intrahealth.org).

Bien que l'accès aux soins de santé préoccupe les autorités de l'Afrique de l'Ouest francophone (AOF) depuis de nombreuses années, ces dernières se sont principalement concentrées sur la question de la proximité géographique. Cependant, la persistance de la faible utilisation des services de santé a mis en évidence les multiples problèmes qui limitent l'accès, notamment les barrières financières. Ce constat se vérifie particulièrement dans les zones urbaines, où de nombreuses personnes n'ont pas accès aux soins, en particulier aux services de planification familiale (PF), malgré leur proximité avec les établissements de soins de santé.[1] Les villes de l'AOF présentent une forte prévalence de la pauvreté, avec des écarts croissants entre les classes, des taux de prévalence contraceptive faibles et des besoins non satisfaits élevés (28 %).[2]

S'appuyant sur le succès avéré de l'Initiative sur la santé reproductive en milieu urbain, The Challenge Initiative (TCI) a été mis sur pied, dans le but d'accélérer l'utilisation des méthodes contraceptives dans les zones urbaines pauvres. Depuis 2016, TCI en AOF, sous la direction d'IntraHealth International, a amélioré l'accès aux services de PF pour les femmes en âge de procréer (15-49 ans) par le biais d'interventions à haut impact, notamment les Journées spéciales de planification familiale (JPSF) adoptées dans le cadre de l'Initiative sur la santé reproductive en milieu urbain. Il ressort de la documentation relative aux interventions menées dans le cadre de ce projet que les objectifs autour des JSPF ont été largement dépassés en termes de recrutement de nouvelles clientes de PF (104 %) et de couples-années de protection (118 %).[3] L'approche des JSPF a également été testée en Mauritanie dans le cadre du projet Agir pour la planification familiale financé par l'Agence américaine pour le développement international en 2017. L'exploitation des documents relatifs à cette approche révèle des résultats exceptionnels enregistrés lors des Journées spéciales par rapport aux jours ordinaires, avec 5,5 client(e)s de plus recrutés et 7 nouveaux client(e)s de plus.[4]Les JSPF ont également été intégrées à TCI University, une plateforme en ligne qui héberge le cadre et les outils associés aux interventions éprouvées de PF et de santé sexuelle et reproductive des adolescents et des jeunes.[5]

Nous décrivons la façon dont les municipalités et le système de santé ont collaboré pour mettre en œuvre les JSPF dans les zones urbaines , l'effectivité de l'accès des femmes aux services de PF, les coûts associés, les leçons apprises et les recommandations pour maintenir et mettre à l'échelle les JSPF.

> A travers les JSPF, les municipalités ont fourni des services de PF gratuits pendant des jours spécifiques au sein de la communauté, dans des établissements de santé ou dans des sites proches des habitations.

Les JSPF

Les JSPF sont en effet une intervention de PF organisée par les municipalités pour fournir des services de PF gratuits pendant des jours spécifiques au sein de la communauté. Chaque édition des JSPF se déroulait sur 2 à 5 jours consécutifs au sein de l'établissement de santé (stratégies fixes) ou dans d'autres sites proches des habitations (stratégies avancées ou mobiles). Cette intervention a été mise en œuvre dans un contexte régional où les établissements de santé publique, à l'exception de ceux du Burkina Faso, offraient habituellement des produits et services contraceptifs payants.

Rôles des différentes parties prenantes

Les villes soutenues par TCI AOF ont intégré les JSPF dans leurs plans d'action dans le but de renforcer la PF ainsi que la santé sexuelle et reproductive des adolescents et des jeunes. Dans le cadre de la mise en œuvre de ces plans, TCI, les municipalités et le système de santé ont créé des Unités de coordination et de gestion des programmes (UCG) composées de points focaux gouvernementaux issus de chaque municipalité (y compris des responsables financiers), des districts de sanitaires et de la direction régionale de la santé. Chaque municipalité a signé un arrêté établissant l'Unité de coordination et de gestion du programme (UCG) et a intégré celle-ci à l'organisation institutionnelle de la ville afin d'assurer la durabilité des UCG.

Préparation et mobilisation des ressources

L'UCG se réunissait tous les mois pour planifier les activités et évaluer les progrès réalisés. Des décisions étaient prises concernant les points suivants : les lieux des JSPF, la population cible (généralement les populations pauvres et défavorisées), le nombre de JSPF à organiser, le personnel requis, la logistique, les produits contraceptifs et consommables nécessaires, et le budget de l'activité. Les termes de référence pour les JSPF et les demandes de budget étaient soumis à la municipalité en vue du déboursement des fonds. Le point focal de TCI avait travaillé avec l'UCG pour s'assurer que le plan d'action de la ville comportant les JSPF, étaient inclus dans les plans de travail annuels des districts sanitaires.

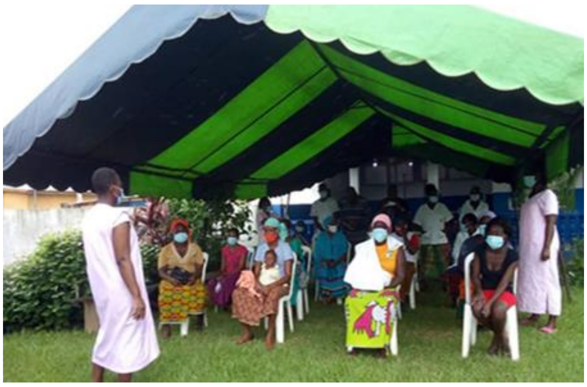

Des membres de la communauté assistent à une séance de sensibilisation sur la planification familiale lors d'une Journée spéciale de la planification familiale pendant la pandémie de la COVID à Abobo, Abidjan, Côte d'Ivoire. 2020 Kouakou Yao Degah Bienvenu Attoumbre/The Challenge Initiative Afrique de l'Ouest francophone-IntraHealth International.

Des réunions préparatoires réunissant les responsables du système de santé, de la municipalité et de la communauté (représentée par un relais communautaire ou le délégué de quartier) étaient organisées afin d'évaluer les besoins en produits contraceptifs et consommables, préparer les outils de gestion (formulaires, registres, fiches, etc.), mobiliser la logistique (véhicules, bâches, chaises, sonorisation) et déterminer le personnel qualifié du système de santé (médecins, sage-femmes, infirmiers) et le personnel d'appui (agents de santé communautaire, chauffeurs, agents d'entretien, etc.). Enfin, une visite préalable du site était effectuée pour vérifier que les locaux (cases de santé, hôtels de ville et centres socioculturels) étaient adéquats.

Marketing et Communication

Un plan de communication pour les JSPF était élaboré et mis en œuvre pendant 4 à 5 jours (2 à 3 jours avant l'activité et les 2 premiers jours de l'activité). Les messages étaient diffusés par le biais des médias de masse (affiches, banderoles, radio, télévision, réseaux sociaux et journaux) et des crieurs publics. Les relais communautaires avaient organisé des réunions d'information avec les chefs religieux, traditionnels et communautaires et avaient effectué des visites à domicile au cours desquelles ils avaient distribué des cartes de référence et donné des informations sur les JSPF.

Déroulement des JSPF

> Lors des JSPF, les prestataires de soins de santé tiennent des causeries éducatives, à la suite desquelles les femmes sont dirigées vers des salles de consultation où on leur [illegible]

Les JSPF en tant qu'approche de proximité étaient organisées dans les établissements de santé (stratégies fixes) ou sur d'autres sites proches des habitations (stratégies avancées ou mobiles). Lors des JSPF, après les causeries éducatives animées par les prestataires de santé à l'intention des femmes, celles-ci étaient dirigées vers des salles de consultation où des conseils leur étaient prodigués, la méthode de PF de leur choix était proposée, et des consignes pour les visites de suivi étaient données. Pour des besoins de suivi et de gestion des complications, les clientes étaient tenues de retourner à l'établissement de santé. Au cas où le personnel du site se voyait incapable de gérer la complication, la cliente était référée vers l'hôpital du district à l'aide d'un formulaire de référence standard. Les JSPF étaient menées sous la supervision de l'équipe de cadre du district sanitaire afin de s'assurer que les services offerts étaient de haute qualité et que l'activité se déroulait sans problème. En 2021, les JSPF ont été organisées dans 10 villes de 5 pays de l'Afrique de l'Ouest Francophone. (Tableau 1).

Gestion des données

Les outils de gestion des JSPF étaient les mêmes que ceux utilisés par le système de santé, notamment les cartes de rendez-vous, les cartes de PF, les registres de PF, les formulaires de référence et de contre-référence, et un formulaire de synthèse des données des JSPF.

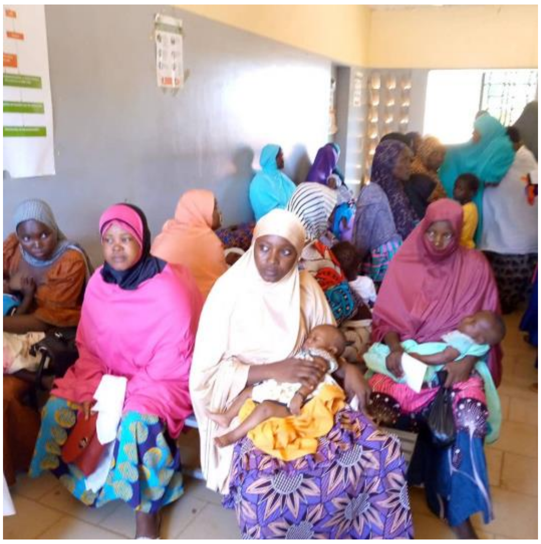

Des femmes participent à une journée spéciale sur la planification familiale à Niamey, au Niger. 2021 Fatimata Sow/The Challenge Initiative Afrique de l'Ouest francophone-IntraHealth International.

Les équipes sur le terrain élaboraient des rapports journaliers et un rapport de synthèse qui étaient ensuite envoyés au district, puis à TCI. Le rapport de synthèse comprenait le lieu, les populations sensibilisées lors des JSPF, le personnel de santé mobilisé, les utilisateurs des services proposés lors des JSPF et les quantités de produits distribués. Chaque mois, les formations sanitaires intégraient ces données dans leur propre rapport mensuel. Le gestionnaire de données du district se chargeait de saisir ces données dans la base de données de TCI AOF et d'archiver les fiches de collecte et les rapports.

MÉTHODES

Cette analyse descriptive, qui couvre la période de juillet 2020 à juin 2021, s'est appuyée sur la revue documentaire des rapports d'activité, l'analyse des données du système d'informations sanitaires et la collecte de données rétrospectives sur les coûts de mise en oeuvre. Tous les établissements de santé ayant organisé des JSPF et dont les données ont été saisies dans la base de données du projet entre juillet 2020 et juin 2021 ont été sélectionnés dans les 36 districts soutenus par TCI. Nous avons élaboré un formulaire de collecte de données sur le logiciel Epi Info 7 pour saisir et analyser les données financières provenant de tous les rapports de dépenses des JSPF de toutes les villes au cours de la période d'étude. Le Tableau 2 présente les indicateurs utilisés pour cette analyse.

Approbation éthique

Cette analyse est conforme aux normes internationales en matière d'éthique de recherche. Aucune information personnelle n'a été collectée. Les questions des informateurs clés avaient trait uniquement au déroulement normal de leur travail.

RÉSULTATS

Caractéristiques des établissements de santé qui mettent en œuvre les JSPF

Au cours de la période analysée, 1 046 JSPF ont été organisées dans 452 établissements de santé. Plus de la moitié (51,2 %) des établissements de santé partenaires de TCI ont organisé des JSPF. Le pourcentage variait considérablement d'une ville à l'autre, de 23,8 % dans l'Union des communes du Zou (UCOZ), au Bénin, à 91 % à Bouaké, en Côte d'Ivoire (Figure 1).
Parmi les établissements de santé ayant organisé des JSPF, 94% étaient du secteur public et 6% du secteur privé. Le nombre moyen de jours d'activités lors des JSPF organisées par établissement de santé dans toutes les villes était de 2,3 jours, la ville de Bouaké avait le nombre de jours le plus élevé (5,1) (Tableau 3).

Caractéristiques du personnel de santé mobilisé pour les JSPF

Les JSPF ont été majoritairement menées par des sage-femmes, avec une moyenne de 221 (42%) sage-femmes par mois. Ces dernières étaient suivies par des agents de santé communautaires, des infirmières et des médecins (Figure 2). Un tel résultat était attendu car les sage-femmes offrent habituellement les services de PF avec le soutien des agents de santé communautaire et des infirmièrs(es). En général, les médecins étaient chargés de la supervision du déroulement des JSPF. Ouagadougou a mobilisé un personnel plus important, tandis que Bouake n'a mobilisé que des sage-femmes. (Figure 3).

TABLEAU 1. Les villes soutenues par TCI qui mettent en œuvre des JSPF en Afrique de l'Ouest francophone, juillet 2020-juin 2021.

Pays	Ville	Taille de la Pop.	Femmes en âge de procréer	Ets. de santé impliqués	Taux de prévalence contraceptive par pays et par région, %[6–10]	Taux de besoins non satisfaits par pays et par région, %[6–10]
Bénin	Abomey Calavi	1 480 982	355 436	63	9,9	37,5
	UCOZ	1 228 667	294 880	116	13,9	31,2
	Cotonou	1 108 320	265 997	73	19,8	35
Burkina Faso	Koudougou	183 332	408 207	67	31,8	16,3
	Ouagadougou	2 415 266	876 104	153	38	16,9
Côte d'Ivoire	Abidjan	5 616 633	2 684 751	155	21,7	21,9
	Bouaké	931 851	145 953	32	21,9	18,6
Niger	Niamey	1 565 056	375 613	62	26,2	17,7
Sénégal	Nioro	365 534	87 728	51	23,6	22,6
	Ziguinchor	319 425	79 856	109	22,6	22,7
Total		15 215 066	5 574 525	881		

Abréviations : TCI, The Challenge Initiative; UCOZ, Union des communes du Zou.

TABLEAU 2. Indicateurs utilisés dans l'analyse des Journées spéciales de planification familiale en Afrique de l'Ouest francophone.

Indicateur	Définition	Source des données
Nombre moyen de jours d'activité de JSPF par établissement de santé	Le nombre total de jours d'activités de JSPF divisé par le nombre total d'établissements ayant accueilli des JSPF	Données du projet
Nombre de personnes sensibilisées aux JSPF	Le nombre de personnes (hommes et femmes) qui étaient au courant des JSPF	Données du SGIS
Nombre total d'utilisateurs recrutés pendant les JSPF	Le nombre total d'utilisateurs nouveaux et anciens de la PF qui ont été servis pendant les JSPF.	Données du SGIS
Volume de clients annuels lors des JSPF	Le nombre total d'utilisateurs (nouveaux et anciens) ajusté pour tenir compte des éléments suivants :<br>• Saisonnalité (sommes glissantes) avec somme glissante sur 12 mois (juillet 2020-juin 2021) pour chaque méthode contraceptive<br>• Nouvelles visites (*revisit*) (pour les méthodes à courte durée d'action) à l'aide de coefficients CAP inversés	Données du SGIS
Rendement quotidien des JSPF ou nombre moyen d'utilisateurs recrutés par jour	Le nombre total d'utilisateurs recrutés lors des JSPF sur le nombre total de jours d'activités des JSPF.	Données du SGIS
Nombre total d'adolescents et de jeunes recrutés dans le cadre des JSPF	Le nombre total de nouvelles et anciennes utilisatrices de PF, âgées de 15 à 24 ans, qui ont été servies dans le cadre des JSPF.	Données du SGIS
Coût moyen des JSPF	Le montant total dépensé pour les JSPF divisé par le nombre total de jours d'activités des JSPF.	Données du projet
Coût opérationnel moyen du recrutement d'une utilisatrice de PF dans le cadre des JSPF	Le montant total dépensé pour les JSPF divisé par le nombre total d'utilisatrices de PF recrutées dans le cadre des JSPF.	Données du projet

Abréviations : CAP, Couples-années de protection ; PF, planification familiale ; JSPF, Journée spéciale de la planification familiale ; SGIS, système de gestion de l'information sanitaire.

FIGURE 1. Pourcentage d'établissements de santé soutenus par The Challenge Initiative ayant organisé des journées spéciales de planification familiale en Afrique de l'Ouest francophone, par ville, juillet 2020-juin 2021.

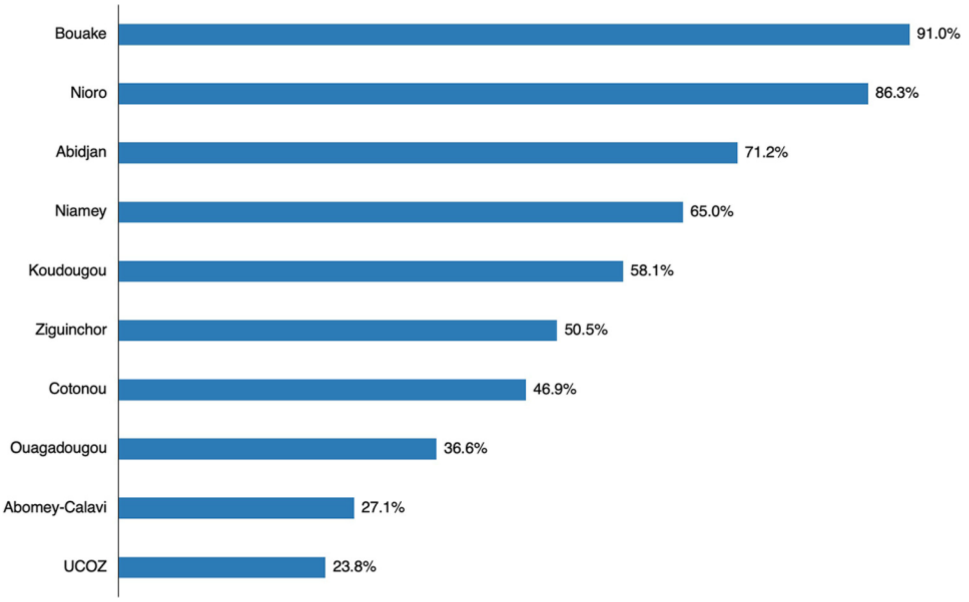

Abréviation : UCOZ, Union des communes du Zou.

TABLEAU 3. Nombre moyen de Journées spéciales de planification familiale en Afrique de l'Ouest francophone par établissement de santé dans les villes, juillet 2020-juin 2021.

Pays	Villes	Établissements de santé ayant accueillis des JSPF	Nombre total de JSPF organisées	Nombre moyen de JSPF organisées par établissement de santé
Côte d'Ivoire	Abidjan	42	56	1,3
	Bouake	15	77	5,1
	Subtotal	57	133	2,3
Benin	UCOZ	76	166	2,2
	Abomey-Calavi	15	46	3,1
	Cotonou	52	173	3,3
	Subtotal	143	385	2,7
Niger	Niamey	36	82	2,3
Burkina Faso	Ouagadougou	56	95	1,7
	Koudougou	61	97	1,6
	Subtotal	117	192	1,6
Senegal	Ziguinchor	55	108	2,0
	Nioro	44	146	3,3
	Sous-total	99	254	2,6
Total		452	1046	2,3

Abréviations : JSPF, Journée spéciale de planification familiale ; UCOZ, Union des communes du Zou.

FIGURE 2. Pourcentage du personnel de santé mobilisé pour les Journées spéciales de planification familiale en Afrique de l'Ouest francophone par catégorie, juillet 2020-juin 2021 (N=525)

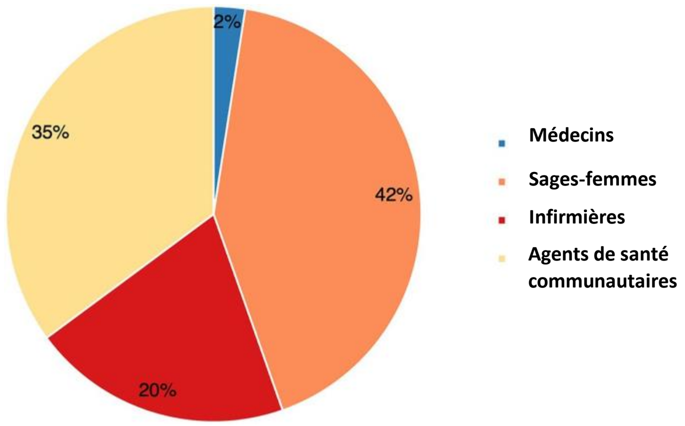

Sensibilisation des populations sur les JSPF

Lors des JSPF, 583 volontaires communautaires (ou agents de santé) ont mené 22 960 activités de sensibilisation. Au total, 1 638 émissions diffusées dans les médias ont touché 181 792 personnes, dont 17 % d'hommes. Parmi les personnes sensibilisées sur les JSPF, la majorité était âgée de 20 à 24 ans (53%) comparée aux personnes âgées de 25 à 49 ans (44%). Niamey, UCOZ et Ouagadougou ont enregistré le plus grand nombre de personnes touchées lors des JSPF. Parmi les personnes touchées, 54 619 (30%) étaient orientées vers les JSPF (données non présentées).

Résultats de la prestation de services

De juillet 2020 à juin 2021, les JSPF ont permis de fournir des services de PF à 71 669 utilisatrices de méthodes contraceptives modernes. Près de la moitié (46%) des utilisatrices de services offerts dans le cadre des JSPF étaient âgés de 15 à 24 ans. Les méthodes contraceptives les plus utilisées étaient les implants, suivies des injectables et des pilules, avec respectivement 34,3 %, 33,9 % et 15,2 % (Tableau 4). Un pourcentage similaire de méthodes à longue durée d'action a été enregistré lors de la mise en œuvre des JSPF en Mauritanie.[4] Ces chiffres peuvent s'expliquer en partie par la gratuité des dites méthodes pendant les jours d'activités des JSPF, ce qui n'est pas souvent le cas lors des jours ordinaires où elles coûtent très chères. Les méthodes à longue durée d'action sont généralement plus chères que les autres méthodes les jours ordinaires dans les établissements de santé. Par exemple, en Côte d'Ivoire, le coût des services courants pour la pose d'un implant, incluant les consommables, est de 3 000 FCFA (5 USD), de 1000 FCFA (1,70 USD) pour les injectables et de 200 FCFA (0,34 USD) pour les pilules, alors qu'au Sénégal, les coûts pour les mêmes services sont inférieurs : 1500 FCFA (2,60 USD) pour les implants, 200 FCFA (0,34 USD) pour les injectables et 100 FCFA (0,17 USD) pour les pilules.

Dans tous les groupes d'âge, 58% des bénéficiaires étaient de nouveaux utilisateurs de contraceptifs. Ce pourcentage était beaucoup plus élevé chez les adolescents et les jeunes, qui représentaient 51% des nouveaux utilisateurs de contraceptifs. Ce résultat montre que l'intervention menée dans le cadre des JSPF a mobilisé de nouvelles utilisatrices grâce à une combinaison de services gratuits, de proximité, de commodité et de sensibilisation.
Ces résultats ont été ajustés afin de prendre en compte la saisonnalité et le couple-années de protection pour les utilisateurs de méthodes à courte durée d'action ; ceci, dans le but de calculer le volume annuel de la clientèle, soit 34 061 utilisateurs (Tableau 5). L'analyse de la combinaison de méthodes a montré que 78 % de ces utilisateurs ont adopté des méthodes à longue durée d'action et 22 % des méthodes à courte durée d'action entre juillet 2020 et juin 2021. Parmi les utilisateurs ayant choisi des méthodes à longue durée d'action, 43 % étaient des adolescents et des jeunes.
Le nombre moyen d'utilisateurs recrutés par jour pendant les JSPF (rendement journalier) était de 68. Ce chiffre est beaucoup plus élevé que celui de la Mauritanie qui ne comptait que 28 utilisateurs recrutés en moyenne par jour.[4] La ville d'Abidjan, avec une moyenne de 222 utilisateurs recrutés par jour, a enregistré la plus grande performance journalière, suivi par l'UCOZ (99 utilisateurs) et Niamey (97 utilisateurs). (Tableau 6). Ces différences peuvent s'expliquer par plusieurs facteurs : (1) la taille de la population (la ville d'Abidjan est la plus peuplée de toutes les villes, suivie de Ouagadougou, Niamey, Abomey Calavi et UCOZ) ; (2) le coût des services de PF courants, comme mentionné ci-dessus ; et (3) le niveau des besoins non satisfaits, qui est le plus

élevé au Bénin (32 %), en Côte d'Ivoire (22 %) et au Sénégal (22 %).[6–10]

FIGURE 3. Nombre d'agents de santé mobilisés pour les Journées spéciales de planification familiale en Afrique de l'Ouest francophone, par catégorie et par ville, juillet 2020-juin 2021.

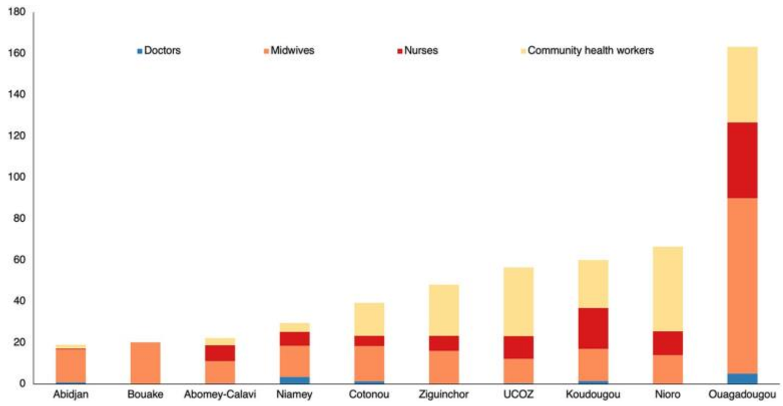

Abréviation : UCOZ, Union des communes du Zou.

TABLEAU 4. Bénéficiaires des Journées spéciales de planification familiale en Afrique de l'Ouest francophone par groupe d'âge et type de méthode, juillet 2020 - juin 2021.

	Util. âgés de 25 à 49 ans, Nbre.		Util. âgés de 25 à 49 ans, Nbre.		Util. âgés de 25 à 49 ans, Nbre.		
Type de méthodes de Contraception	Nouveaux d'ut	Anciens	Nouveaux d'ut	Anciens	Nouveaux d'ut	Anciens	Nbre total, (%)
Implants	2926	712	5243	2135	8708	4833	24 557 (34,3)
Injectable	2035	1,032	4000	3532	6285	7414	24 298 (33,9)
Pilule	1058	817	1588	1954	2015	3433	10 865 (15,2)
Condoms	1817	656	1603	897	1487	1172	7632 (10,6)
DIU	188	74	567	241	1885	1195	4150 (5,8)
Contraceptif d'urgence	20	14	42	25	34	32	167 (0,2)
Total	8044	3305	13 043	8784	20 414	18 079	71 669 (100)

Abréviation : Stérilet, dispositif intra-utérin.

TABLEAU 5. Volume de clients annuel de la planification familiale pendant les Journées spéciales de planification familiale en Afrique de l'Ouest francophone, par groupe d'âge et par catégorie de méthode, juillet 2020 - juin 2021.

Méthode de contr.	Nbre d'ut. âgés de 15 à 19 ans	Nbre d'ut. âgés de 20 24 ans.	Nbre d'ut. âgés de 25 à 49 ans.	Nbre total d'ut. (%)
À longue durée d'action	3587	7606	15 227	26 420 (78)
À courte durée d'action	1188	2404	4049	7641 (22)
Total	4775	10 010	19 276	34 061 (100)

Le nombre d'établissements de santé ayant organisé des JSPF et le nombre de jours d'activités de JSPF n'ont pas eu d'incidence considérable sur le rendement des JSPF. La ville d'Abidjan, qui a organisé 56 jours d'activités de JSPF dans 42 établissements de santé, a eu un rendement plus élevé que Koudougou, Cotonou, Nioro ou Ziguinchor, où les JSPF ont été organisées sur un plus grand nombre de jours et dans un peu plus d'établissements de santé.

En outre, le nombre moyen global de clients servis par jour et par prestataire (médecins, sage-femmes et infirmières) était de 0,2, avec des variations significatives selon les villes. La ville d'Abidjan a enregistré le plus

grand nombre de clients servis par jour et par prestataire (13,1), et les villes de Koudougou et Ouagadougou ont enregistré le plus petit nombre de clients servis par prestataire (0,7)) (Tableau 6). Le faible nombre d'utilisateurs servis par les prestataires dans certaines villes (Cotonou, Nioro ou Ziguinchor) peut refléter la faible affluence lors des Journées spéciales qui est souvent due à un manque de préparation (par exemple, insuffisance de la communication). Pour les villes du Burkina Faso, ce fait pourrait être lié au contexte national notamment à la gratuité des services de planification familiale.

Nos résultats montrent que l'intervention menée dans le cadre des JSPF a mobilisé de nouveaux utilisateurs grâce à une combinaison de services gratuits, de proximité, de commodité et de sensibilisation.

Coûts de mise en oeuvre

Dans l'ensemble, les JSPF en AOF ont coûté 145 382 501 FCFA (252 839 USD). Les contributions provenaient principalement des municipalités (51 223 176 FCFA [34,9%]) et de TCI AOF (94 159 325FCFA [64,8%]), et le reste est venu des comités de gestion des établissements de santé composés de membres de la communauté. La ville de Niamey, qui a supporté 53% des dépenses (11 735 400 FCFA), est la seule ville à avoir dépensé plus que TCI. En dehors de Niamey, Cotonou, Abomey Calavi, Abidjan et Bouaké ont enregistré des contributions supérieures à la moyenne, avec respectivement 5 046 800 FCFA (44%), 2 279 720 FCFA (41%), 9 671 200 FCFA (39%) et 6 090 000 FCFA (39%) respectivement (Tableau 7). Ouagadougou et Koudougou ont enregistré les contributions les plus faibles car les produits contraceptifs étant gratuits au Burkina Faso, les dépenses n'étaient effectuées qu'en cas de pénurie de produits contraceptifs ou de consommables.

TABLEAU 6. Rendement journalier des Journées spéciales de planification familiale par ville en Afrique de l'Ouest francophone, juillet 2020 - juin 2021

Pays	Ville	Nbre total d'ut. recrutés lors des JSPF	Nbre total de JSPF.	Nbre moyen d'ut. recrutés par JSPF	Nbre total de prestataires	Nbre d'ut. recrutés par JSPF par prestataire.
Côte d'Ivoire	Abidjan	12 450	56	22 2	17	13,1
	Bouake	5850	77	76	20	3,8
	Subtotal	18 300	133	13 8	37	3,7
Benin	UCOZ	16 454	166	99	23	4,3
	Abomey-Calavi	3784	46	82	19	4,3
	Cotonou	4131	173	24	23	1,0
	Sous-total	24 369	385	63	65	1,0
Niger	Niamey	7947	82	97	25	3,9
Burkina Faso	Ouagadougou	8290	95	87	127	0,7
	Koudougou	2065	97	21	37	0,6
	Sous-total	10 355	192	54	164	0,3
Sénégal	Ziguinchor	5338	108	49	23	2,1
	Nioro	5360	146	37	26	1,4
	Sous-total	10 698	254	42	49	0,9
Total		71 669	1,046	68	340	0,2

Abréviations: JSPF, Journées spéciales de planification familiale; UCOZ, Union des communes du Zou.

TABLEAU 7. Répartition des contributions financières aux Journées spéciales de planification familiale en Afrique de l'Ouest francophone par Ville et Source, Juillet 2020–Juin 2021

Pays	Ville	Contribution par ville, FCFA (%)	Contribution TCI, FCFA (%)	Contribution totale, FCFA
Côte d'Ivoire	Abidjan	9 671 200 (39)	15 274 000 (61)	24 945 200
	Bouaké	6 090 000 (39)	9 450 000 (61)	15 540 000
	Sous-total	15 761 200 (39)	24 724 000 (61)	40 485 200
Bénin	UCOZ	4 500 000 (32)	9 652 350 (68)	14 152 350
	Abomey-Calavi	2 279 720 (41)	3 343 200 (59)	5 622 920
	Cotonou	5 046 800 (44)	6 474 400 (56)	11 521 200
	Sous-total	11 826 520 (38)	19 469 950 (62)	31 296 470
Niger	Niamey	11 735 400 (53)	10 350 000 (47)	22 085 400
Burkina Faso	Ouagadougou	850 000 (5)	15 550 000 (95)	16 400 000
	Koudougou	1 539 991 (23)	5 050 000 (77)	6 589 991
	Sous-total	2 389 991 (10)	20 600 000 (90)	22 989 991
Sénégal	Ziguinchor	6 663 265 (37)	11 585 375 (63)	18 248 640
	Nioro	2 846 800 (28)	7 430 000 (72)	10 276 800
	Sous-total	9 510 065 (33)	19 015 375 (67)	28 525 440
Total		51 223 176 (35)	94 159 325 (65)	145 382 501

Abréviations : FCFA : Franc CFA de l'Afrique de l'Ouest. TCI : The Challenge Initiative. UCOZ : Union des Communes du Zou.
[a] 611 FCFA en Afrique de l'Ouest =1USD.

Les dépenses de TCI ont été consacrées au marketing et à la communication, à la logistique et aux indemnités journalières du personnel. La majeure partie de la contribution des municipalités a été consacrée à l'achat de produits contraceptifs (90,8 %). Les indemnités journalières du personnel représentaient 33,8% des dépenses, les produits contraceptifs venaient en seconde position avec 31,7% (Figure 4).
Le coût moyen pour un seul jour d'activité des JSPF en AOF était de 138 989 FCFA (242 $ US). Les coûts moyens les plus élevés pour un seul jour d'activité des JSPF ont été enregistrés à Abidjan, Bouaké et Niamey. Les villes de Cotonou et Koudougou ont quant à elles enregistré le coût moyen le plus bas pour un seul jour d'activité des JSPF (Tableau 8). Les différences enregistrées dans les taux d'indemnités journalières et les catégories de personnel mobilisé pour les JSPF, notamment à Abidjan et Bouaké, où près de 85% du personnel étaient des sage-femmes, pourraient expliquer cette variation.

Le coût moyen de recrutement d'une utilisatrice de PF était de 2029 FCFA (4 USD) de juillet 2020 à juin 2021. Ce coût moyen variait de 860 FCFA (1 USD) pour l'UCOZ à 3419 FCFA (6 USD) pour Ziguinchor (Tableau 8). Les différences notées dans les performances des villes en matière de recrutement d'utilisatrices de PF dans le cadre des JSPF n'étaient pas liées au montant des dépenses. En effet, les villes de Koudougou et de Ziguinchor avaient le coût moyen de recrutement par utilisatrice de PF le plus élevé par rapport à Abidjan et à l'UCOZ qui avaient plus de succès en termes d'utilisatrices recrutées et avaient des coûts moyens par utilisatrice de PF plus faibles. En termes de couples-années de protection du couple, le coût moyen par utilisatrice des JSPF était 4268 FCFA (7 USD).

Les JSPF contribuent à lever les obstacles économiques qui empêchent certaines femmes d'accéder aux méthodes contraceptives, en particulier dans les zones urbaines pauvres.

LEÇONS TIRÉES

- Les JSPF ont amélioré l'accès des femmes en âge de procréer à la contraception, tant sur le plan géographique que financier. Grâce à la fourniture de services de PF gratuits, les JSPF ont permis à 71 669 femmes en âge de procréer d'accéder à des méthodes contraceptives modernes dans les zones urbaines en AOF, avec un volume annuel de clients de 34 061. En Mauritanie, le nombre de clients servis par jour pendant les JSPF était 5,5 fois plus élevé que celui enregistré lors des services habituels payants, avec 6 nouveaux clients et 21 anciens clients .

- Les JSPF ont permis aux clients de bénéficier gratuitement de méthodes à longue durée d'action. Sur le volume annuel de clientes enregistrées pendant les JSPF, la majorité (78 %) des utilisatrices ont adopté des méthodes à longue durée d'action et seulement 22 % ont adopté des méthodes à courte durée d'action. Une tendance similaire existe presque partout en Afrique subsaharienne, où l'utilisation d'implants a augmenté de manière substantielle et uniforme dans presque toutes les catégories sociodémographiques.[11]
- Les JSPF ont renforcé la collaboration entre les parties prenantes. Nous avons constaté une forte appropriation par les municipalités et le système de santé, qui a permis de mettre en place l'UCGP. Cet organe a réussi à renforcer les collaborations en rapprochant les municipalités et le système de santé. Cette collaboration impliquait plusieurs acteurs : médecins (2%), sage-femmes (42%) et infirmières (20%) ; agents de santé communautaire (35%) ; représentants municipaux ; et la population en général.
- L'intervention dans le cadre des JSPF s'est avérée très rentable. La fourniture de services de contraception complets et de qualité à un nombre croissant de femmes qui ont besoin de services modernes a eu d'importantes implications en termes de coûts.[12] Le coût moyen pour l'organisation d'un seul jour d'activités des JSPF était de 138 989 FCFA (242 USD), et le coût moyen pour le recrutement d'une utilisatrice de PF était de 2029 FCFA (4 USD). En 2012, le coût annuel moyen par utilisatrice pour toutes les méthodes modernes dans le monde en développement était estimé à 6,15 USD et à 11,26 USD pour la région Afrique.[12]

FIGURE 4. Catégories de dépenses pour les journées spéciales de planification familiale en Afrique de l'Ouest francophone, juillet 2020-juin 2021.

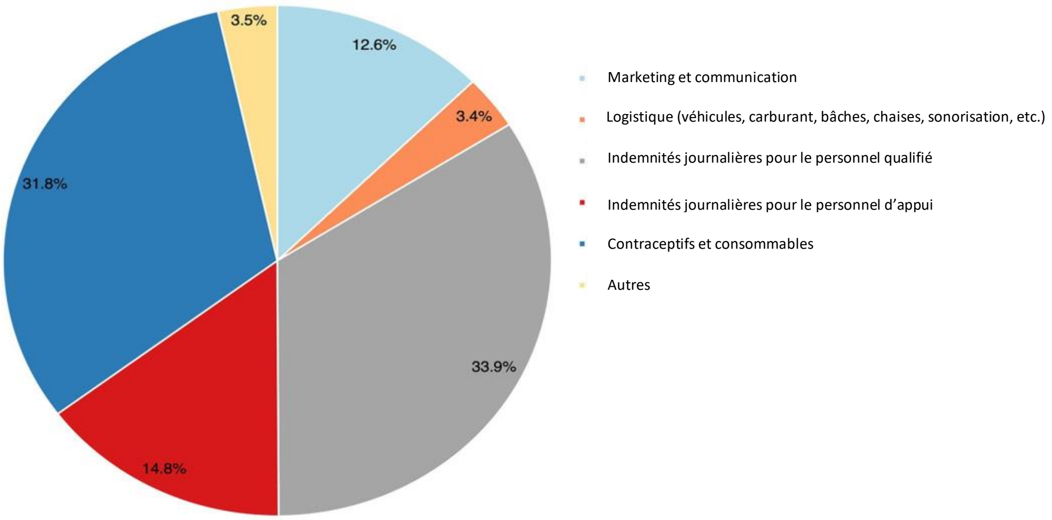

TABLEAU 8. Coût moyen pour un seul jour d'activités des JSPF par ville, juillet 2020 à juin 2021.

Villes	Coût total des JSPF, FCFA[a]	Nbre total des JSPF	Utilisateurs recrutés lors des JSPF	Coût moyen des JSPF, FCFA (US$)	Coût moyen par utilisateur, FCFA (US$)
Abidjan	24 945 200	56	12 450	445 450 (775)	2004 (3)
Bouaké	15 540 000	77	5850	201 818 (351)	2656 (5)
Abomey-Calavi	5 622 920	46	3784	122 237 (213)	1486 (3)
UCOZ	14 152 350	166	16 454	85 255 (148)	860 (1)
Cotonou	11 521 200	173	4131	66 596 (116)	2789 (5)
Niamey	22 085 400	82	7947	269 334 (468)	2779 (5)
Ouagadougou	16 400 000	95	8290	172 631 (300)	1978 (3)
Koudougou	6 589 991	97	2065	67 938 (118)	3191 (6)
Nioro	10 276 800	146	5360	70 389 (122)	1917 (3)
Ziguinchor	18 248 640	108	5338	168 968 (295)	3419 (6)
Total	145 382 501	1046	71 669	138 989 (242)	2029 (4)

Abréviations : FCFA : franc CFA d'Afrique de l'Ouest. PF : planification familiale. JSPF : Journée spéciale de planification familiale. UCOZ : Union des communes du Zou.

[a] 611 FCFA d'Afrique de l'Ouest=1 USD.

RECOMMANDATIONS

Les résultats prometteurs des JSPF en Afrique de l'Ouest Francophone permettent de formuler les recommandations suivantes :

- Les ministères de la santé doivent veiller à la disponibilité des produits contraceptifs et des consommables afin de réduire de manière significative les coûts liés à la mise en œuvre de cette intervention et de permettre aux municipalités de couvrir les coûts restants, tels que les indemnités journalières et la logistique.
- Les ministères de la santé devraient inclure les JSPF dans leurs plans stratégiques de PF afin de permettre aux districts sanitaires de les intégrer dans leurs plans de travail annuels.
- Les membres des unités de coordination et de gestion, ainsi que les groupes de plaidoyer, doivent inciter les municipalités à inclure une ligne budgétaire substantielle pour la PF dans leurs budgets annuels afin d'assurer la durabilité de cette intervention.
- Le ministère de la santé devrait renforcer les capacités de communication des relais communautaires et des prestataires en matière de PF afin de consolider les activités visant à lever les obstacles à la PF, à susciter ou à accroître la demandepour la PF et à renforcer les normes et les valeurs positives.[13]

CONCLUSION

Les JSPF peuvent améliorer l'accès aux services de PF tant sur le plan géographique que sur le plan financier. Les résultats des JSPF en Afrique de l'Ouest francophone étaient encourageants, malgré la pandémie de la COVID, grâce au soutien financier des municipalités impliquées et de TCI. Une mise en œuvre réussie nécessite la coordination et l'engagement de toutes les parties prenantes. Ces résultats devraient inciter les municipalités et les systèmes de santé à institutionnaliser l'intervention JSPF en l'intégrant dans les politiques et les normes nationales, en améliorant la disponibilité des produits contraceptifs et des consommables, et en continuant à fournir des financements.

Remerciements : Les auteurs remercient tous ceux qui ont contribué à la collecte des données, notamment les responsables des programmes pays et les responsables de programme ville de The Challenge Initiative (TCI), toutes les collectivités locales soutenues par TCI et les ministères de la Santé du Bénin, du Burkina Faso, de la Côte d'Ivoire, du Niger et du Sénégal.

Financement : The Challenge Initiative et cette étude ont été soutenus par la Fondation Bill & Melinda Gates dans le cadre de l'Opportunity/Contract ID : OPP1145051.

Avertissement : Le bailleur de fonds n'a joué aucun rôle dans la conception et la mise en œuvre de l'étude, ni dans la décision de la publier.

Contributions des auteurs : MK, HT, SNB, RJN et CID ont contribué à la conception de l'étude et à la collecte des données ; MK, RJN et CID ont contribué au codage et à l'analyse des données ; tous les auteurs ont contribué à l'interprétation des résultats et à la préparation de l'article. Tous les auteurs ont revu et approuvé la version finale de l'article.

Intérêts divergents : Aucun déclaré.

RÉFÉRENCES

1. Nikiema A, Rossier C, Millogo R, Ridde V. Inégalités de l'accès aux soins en milieu urbain africain: le cas de la périphérie nord de Ouagadougou. Présenté à la 6ème Conférence sur la population africaine, du 5 au 9 décembre, 2011, Ouagadougou, Burkina Faso.
2. Partenariat de Ouagadougou. *Rapport général de la 10ème Réunion Annuelle du Partenariat de Ouagadougou, Ouagadougou, du 13 au 16 Décembre, 2021*. Partenariat de Ouagadougou ; 2021.
3. Initiative Sénégalaise de Santé Urbaine (ISSU). *A Family Planning Project in Poor Urban Areas: Report on the Capitalization of Approaches*. Report in French. ISSU;2014. Consulté le 29 janvier 2023. https://tciurbanhealth.org/wp-content/uploads/2017/06/ Rapport-de-capitalisation-ISSU-2014.pdf
4. Agir Pour La Planification Familiale (AgirPF).

Innovative Strategy to Increase Adherence to Family Planning (FP) in Mauritania. Project Summary No. 10. AgirPF/EngenderHealth; 2018.

5. TCI University fact sheet. Consulté le 29 janvier 2023. https:// tciurbanhealth.org/project/tci-university-fact-sheet/

6. Institut National de la Statistique et de l'Analyse Économique (INSAE) et ICF. *Bénin Cinquième Enquête Démographique et de Santé au Bénin (EDSB-V) 2017-2018: Indicateurs Clés, Rapport Préliminaire*. INSAE/ICF; 2018.

7. INSD et ICF. *Enquête Démographique et de Santé du Burkina Faso 2021*. INSD/ICF; 2022. Consulté le 10 janvier 2023. https:// dhsprogram.com/pubs/pdf/PR139/PR139.pdf

8. Institut National de la Statistique-INS et ICF. *Enquête Démographique et de Santé de Côte d'Ivoire, 2021*. INS/Côte d'Ivoire/ICF; 2022. Consulté le 10 janvier 2023. https:// dhsprogram.com/pubs/pdf/PR140/PR140.pdf

9. Institut National de la Statistique (INS) et ICF International. *Enquête Démographique et de Santé et à Indicateurs Multiples du Niger 2012*. INS/ICF International; 2013. Consulté le 10 janvier 2023. https://dhsprogram.com/pubs/pdf/fr277/fr277.pdf

10. Agence Nationale de la Statistique et de la Démographie (ANSD) [Sénégal] et ICF. *Sénégal: Enquête Démographique et de Santé Continue (EDS-Continue 2019)*. ANSD/ICF; 2019. Consulté le 10 janvier 2023. https://dhsprogram.com/pubs/pdf/FR368/ FR368.pdf

11. Jacobstein R. Liftoff: the blossoming of contraceptive implant use in Africa. *Glob Health Sci Pract.* 2018;6(1):17–39. CrossRef. Medline

12. Singh S, Darroch JE. *Adding It Up: Cost and Benefits of Contraceptive Services—Estimates for 2012.* Guttmacher Institute; 2012. Consulté le 10 janvier 2023. https://www.guttmacher.org/ sites/default/files/pdfs/pubs/AIU-2012-estimates.pdf

13. PATH, United Nations Population Fund (UNFPA). *Meeting the Need: Strengthening Family Planning Programs.* PATH/UNFPA; 2006. Consulté le 10 janvier 2023. https://www.unfpa.org/sites/default/ files/resource-pdf/family_planning06.pdf

Révision par les pairs

Reçu : 28 octobre 2022 ; Accepté : 27 décembre 2022 ; Première publication en ligne : 13 février 2023.

Citer cet article comme suit: Kandji M, Talla H, Nakoulma RJF, et al. Increasing contraceptive use through free Family Planning Special Days in poor urban areas in francophone West Africa. *Glob Health Sci Pract*. 2023;11(Suppl 1):e2200227. https://doi.org/10.9745/GHSP-D-22-00227

© Kandji et al. Le présent article est un article à libre accès distribué selon les termes du Creative Commons Attribution 4.0 International License (CC BY 4.0), qui autorise l'utilisation, la distribution et la reproduction sans restriction sur n'importe quel support, à condition que l'auteur original et la source soient correctement cités. Pour consulter une copie de la licence, consultez le site https://creativecommons.org/licenses/by/4.0/. Pour accéder à cet article, suivez le lien permanent suivant: https:// doi.org/10.9745/GHSP-D-22-00227
